# Block Training With Moderate‐ or High‐Intensity Intervals Both Improve Endurance Performance in Well‐Trained Cyclists

**DOI:** 10.1002/ejsc.70067

**Published:** 2025-10-30

**Authors:** Bent R. Rønnestad, Ingvill Urianstad Odden, Kristoffer Schulz Solum, Anne Mette Rustaden, Håvard Hamarsland, Daniel Hammarström, Håvard Nygaard, Knut Sindre Mølmen

**Affiliations:** ^1^ Section for Health and Exercise Physiology University of Inland Norway Lillehammer Norway

**Keywords:** cycling performance, endurance training, high‐intensity interval training, threshold training, training intensity

## Abstract

The purpose of this study was to compare the effects of a 1‐week block of moderate‐intensity interval training (MIT) and high‐intensity interval training (HIT) in well‐trained cyclists. Cyclists (♀ = 1; ♂ = 21; maximal oxygen uptake (V̇O_2max_) = 69.5 (6.0) mL·min^−1^·kg^−1^) performed both a MIT block involving 6 sessions over 7 days (5–7 × 10–14‐min work intervals, rate of perceived exertion (RPE): 14.5 (0.3)) and a HIT block involving 5 sessions over 6 days (5 × 8.75‐min with multiple short intervals, RPE: 17.1 (0.4)). Post‐testing was performed after 6 days of active recovery, and blocks were separated by 2 months. Testing included 15‐min maximal cycling power (PO_15min_), 10 s maximal sprint power (PO_10sec_), and power output at a blood lactate concentration of 4 mmol·L^−1^ (PO_4mmol_). Both the MIT and HIT block improved PO_15min_ (4.9 (8.7)% and 2.8 (5.3)%, respectively), with no difference between blocks (*p* = 0.44). MIT displayed greater improvement than HIT in PO_4mmol_ (4.5 (4.5)% vs. 2.1 (2.7)%, respectively, *p* = 0.03, moderate effect size (ES)), while HIT had a moderate ES compared to MIT for improvement in PO_10sec_ (1.5 (3.7)% vs. −1.5 (7.3)%, respectively), that was not significant (*p* = 0.08). No differences were observed between blocks in changes in V̇O_2max_ (*p* = 0.43) or measures of gross efficiency (*p range* = 0.43–0.79). However, MIT induced a larger increase in % of V̇O_2max_ at PO_4mmol_ compared to HIT (1.2 (3.4)%‐points vs. −0.9 (2.5)%‐points, respectively, *p* = 0.02, moderate ES). In conclusion, both a MIT block (lower work interval intensity but longer work duration) and a HIT block (higher work interval intensity but shorter work duration) can improve endurance performance determinants and PO_15min_ with some work intensity‐specific adaptations.

## Highlights

- This study investigated the effects of a short moderate‐intensity interval training (MIT) block (lower exercise intensity but longer work intervals) compared to a short high‐intensity interval training (HIT) block (higher exercise intensity but shorter work intervals) within well‐trained cyclists.
- Both a 1‐week MIT block and a 1‐week HIT block followed by a 6‐day active recovery period effectively improve endurance performance determinants and 15‐min maximal power output

## 1 Introduction

The endurance training literature commonly divides the exercise‐intensity continuum into three distinct zones: (1) low‐intensity, encompassing workloads below the first lactate threshold, categorized as the moderate domain; (2) moderate‐intensity, comprising workloads between the first lactate threshold and critical power/maximal lactate steady state/second lactate threshold, recognized as the heavy domain; and (3) high‐intensity, involving workloads surpassing critical power/maximal lactate steady state/second lactate threshold, acknowledged as the severe domain (Stöggl and Sperlich 2015; Burnley and Jones 2007). Although cyclists frequently incorporate moderate‐intensity interval training (MIT), defined here as intervals performed in the heavy domain, into their training (Lucía et al. 2000; Zapico et al. 2007; Leo et al. 2020), the efficacy of this type of training, as well as the optimal training volume within this domain, remains a topic of ongoing debate (Burnley et al. 2022; Foster et al. 2022). It is assumed that the adaptive signal of interval training is influenced by both exercise intensity and the accumulated duration of work intervals, highlighting the interaction between these factors rather than their independent effects (Seiler et al. 2013). From this, one could hypothesize that MIT, with its prolonged duration and larger total energy turnover compared to high‐intensity interval training (HIT), may elicit similar training adaptations as HIT.

Accordingly, when cyclists performed four times longer work duration with ∼MIT (4 × 16‐min) than HIT (4 × 4‐min), comparable adaptations in lactate threshold power output and mean power output during the last minute of the maximal oxygen uptake (V̇O_2max_) test (PO_V̇O2max_) occurred after 4–7 weeks (Seiler et al. 2013; Sylta et al. 2017). Additionally, in runners, similar improvements in time to exhaustion were observed when continuous moderate‐intensity sessions were performed with twice the duration of HIT and approximately 70% higher energy turnover (Jarstad and Mamen 2019). Yet, there was a numerical advantage for improvement in the workload associated with V̇O_2max_ for HIT compared to the continuous moderate‐intensity exercise (∼12 vs. ∼3%, respectively). Altogether, the numerical changes in the abovementioned studies indicate work intensity‐specific adaptations (i.e., greater numerical changes in threshold measures after moderate‐intensity interventions and greater numerical changes in PO_V̇O2max_ after high‐intensity interventions). However, when moderate‐intensity and HIT are matched for total energy turnover, HIT appears to elicit greater adaptations in an endurance‐trained population (Helgerud et al. 2007).

To further enhance the performance of already well‐trained endurance athletes, block periodization has been proposed as particularly effective (Issurin 2016). Specifically, it has been observed that a 1‐week HIT block with five HIT sessions can improve PO_V̇O2max_ by ∼3%–5% (Rønnestad et al. 2021; Rønnestad and Vikmoen 2019; Rønnestad, Bjerkrheim, et al. 2022; Solli et al. 2025). Interestingly, it was also recently observed that a 1‐week block with six MIT sessions induced superior performance adaptations compared to regular training in well‐trained cyclists (Mølmen et al. 2025). However, the direct comparison between a 1‐week HIT block and a 1‐week MIT block with a larger overall training volume but lower exercise intensity remains unexplored within well‐trained endurance athletes, and is thus warranted (Mølmen et al. 2025; Norte et al. 2024). To our knowledge, the only study investigating the effects of a moderate‐intensity exercise block versus a HIT block involved moderately trained soldiers, where one group performed five sessions per week of 30‐min continuous running at moderate‐intensity and the HIT group performed five sessions of 4 × 5‐min intervals (Borszcz et al. 2022). Notably, all participants within each group exercised at the same absolute velocity, leading to individual differences in actual training intensity. Still, both groups improved, and also here, there seemed to be work intensity‐specific adaptations favoring the moderate‐intensity group in threshold measurements and the HIT group in V̇O_2max_ (Borszcz et al. 2022). Interestingly, the duration of time ≥ 90% of V̇O_2max_ during interval sessions in a HIT block has been observed to be linked with improvements in V̇O_2max_ (Rønnestad, Bjerkrheim, et al. 2022). Yet, the importance of time ≥ 90% of V̇O_2max_ for adaptations to a MIT block remains to be investigated.

The primary purpose of this study was to compare the effects of a 1‐week MIT block to a 1‐week HIT block in well‐trained cyclists. Secondarily, the relationship between time spent ≥ 90% of V̇O_2max_ and training adaptations during both blocks was investigated. We hypothesized that (1) the MIT block would induce favorable adaptations in lactate threshold measurements, while the HIT block would induce favorable adaptations in measurements around V̇O_2max_ intensity, with both blocks similarly improving surrogate measures of endurance performance, and (2) time ≥ 90% of V̇O_2max_ would be more strongly related to training adaptations in the HIT block than in the MIT block.

## 2 Methods

### 2.1 Study Ethics and Participants

Prior to inclusion all participants provided written informed consent to participate in the study, which followed the Helsinki Declaration of 1975 and was approved by the local ethical committee at the University of Inland Norway (reference ID: Case 10–2022) and the Norwegian Agency for Shared Services in Education and Research (Sikt); reference ID: 351304.

Forty‐three cyclists (♂ = 39 and ♀ = 4) initially volunteered to participate in the study. In total, 21 cyclists withdrew from the study due to respiratory tract infections (*n* = 8), injury (*n* = 1), heavy legs (*n* = 1), excessive total load (*n* = 2), not completing the test protocol (*n* = 1), and other reasons unrelated to the study (*n* = 8). Of these, seven withdrew during the MIT block, 12 between blocks, and two during the HIT block. In total, 22 participants completed the study (♀, 1; ♂, 21; age, 19.2 (3.6) years; body mass, 70.8 (7.9) kg; body height, 181 (7) cm; V̇O_2max_, 69.5 (6.0) mL·min^−1^·kg^−1^). Based on their initial V̇O_2max_, the participants were categorized as performance level 3 (*n* = 3), 4 (*n* = 8), and 5 (*n* = 11), equaling to “trained,” “well‐trained,” and “professional,” respectively (De Pauw et al. 2013; Decroix et al. 2016). The participants had recently finished their off‐season training at the time of inclusion and had an average competitive history of 4.4 (2.7) years.

### 2.2 Experimental Design

**FIGURE 1:**
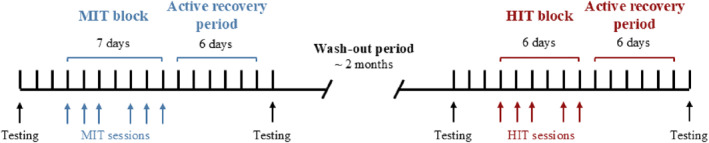
Outline of the study design. First, the participants performed a 7‐day moderate‐intensity training (MIT) block followed by a 6‐day recovery period. After a ∼2‐month wash‐out period with regular training, the participants performed a 6‐day high‐intensity training (HIT) block followed by a 6‐day recovery period. Physiological testing was performed ∼2 days before and 7 days after each block.

This study was preregistered (Mølmen et al. 2022) and utilized a within‐subject, two‐period, two‐treatment design, with 55.1 (16.5) days of regular training between blocks (Figure 1, note the fixed sequence of interventions with the MIT block preceding the HIT block). The present analysis represents a secondary aim of the broader study; thus, no specific a priori sample size calculation was conducted for this comparison. Both training blocks were followed by 6 days of active recovery before post‐testing and all test days were preceded by 2 days of standardized training. Before the first interval session in each block, participants performed 2 days of standardized training. Testing included a prolonged cycling test battery (Rønnestad, Urianstad, et al. 2022) and an isokinetic unilateral knee‐extension test.

### 2.3 Training Periods

The MIT block comprised one daily MIT session over three consecutive days, one rest day, and one daily MIT session over the next three consecutive days (all work intervals performed with a RPE of 14–15 on the Borg 6‐20 scale (Borg 1998)). The first and fourth MIT sessions were performed as 7 × 10‐min work intervals (2.5‐min recoveries), the second and sixth sessions were performed as 6 × 12‐min work intervals (3‐min recoveries), and the third and fifth sessions were performed as 5 × 14‐min work intervals (3.5‐min recoveries). The HIT block comprised one daily HIT session over three consecutive days, one rest day, and one daily HIT session over the two consecutive days. All HIT sessions were performed as 5 × 8.75‐min work intervals consisting of multiple short intervals. During the multiple short intervals participants were instructed to adjust their power output dynamically during each interval to correspond to an RPE of 16–18 on the Borg 6‐20 scale. The guiding of the multiple short intervals was 30 s at ∼118% of 40‐min maximal sustainable power output interspersed with 15 s at ∼60% of 40‐min maximal sustainable power output for 8.75 min, and 3‐min recoveries between work intervals. All participants were experienced cyclists who regularly used the Borg 6‐20 scale in their training. The total work interval duration was 7 h and 4 min in the MIT block and 3 h and 42 min in the HIT block.

In both training blocks, sessions were initiated by a standardized 15‐min progressive warm‐up protocol, followed by 5 min of rest before initiating the first work interval. All sessions in both blocks were supervised by a test leader assessing the participants’ RPE immediately after each work interval. Power output and heart rate (HR) were continuously monitored during all work intervals using the participants’ personal power meters (Tacx Neo 2/Wahoo Kickr/CompuTrainer) and HR monitors. During the second and last interval session in both training blocks, V̇O_2_ and respiratory exchange ratio (RER) were continuously measured (10 s sampling time) during all work intervals using a computerized metabolic system with mixing chamber (Vyntus CPX, Jaeger‐CareFusion, UK) that was calibrated before every session. The average aerobic energy turnover during work intervals in kilojoules per sec (kJ·s^−1^) was calculated using the average V̇O_2_ and RER during work intervals and the energetic equivalents of O_2_ (Péronnet and Massicotte 1991) according to the constants proposed by Kipp et al. (2018). During these sessions, blood lactate concentration ([La^−^]) was also measured immediately after each work interval (Biosen C‐line Lactate Analyzer; EKF Diagnostic GmbH, Barleben, Germany; Table 1).

**TABLE 1:** Training load during the interval sessions in the moderate‐intensity training (MIT) block and high‐intensity interval training (HIT) block, respectively.

Session	MIT block							HIT block						Block *p*
	1	2	3	4	5	6	Average	1	2	3	4	5	Average	
Type of session	7 × 10 min	6 × 12 min	5 × 14 min	7 × 10 min	5 × 14 min	6 × 12 min		5 × 8.75 min	5 × 8.75 min	5 × 8.75 min	5 × 8.75 min	5 × 8.75 min		
Aerobic energy turnover (kJ)		5954 (708)				5992 (752)	**5973 (717)**		3947 (492)			4005 (461)	**3976 (469)**	< 0.01
Aerobic energy turnover (kJ·s^−1^)		1.38 (0.16)				1.39 (0.17)	**1.38 (0.17)**		1.50 (0.19)			1.53 (0.18)	**1.51 (0.18)**	< 0.01
V̇O_2_ (mL·min^−1^·kg^−1^)		53.8 (4.7)				54.2 (4.8)	**54.0 (4.7)**		57.9 (6.5)			58.8 (6.6)	**58.4 (6.5)**	< 0.01
% of V̇O_2max_		77.9 (5.0)				78.4 (4.0)	**78.1 (4.4)**		82.8 (4.4)			84.0 (3.5)	**83.4 (4.0)**	< 0.01
Time ≥ 90% of V̇O_2max_ (m:s)		05:24 (09:39)				04:03 (04:06)	**04:44 (07:18)**		09:29 (07:32)			11:49 (08:03)	**10:39 (07:44)**	< 0.01
HR (bpm)	165 (10)	168 (7)	165 (13)	169 (9)	168 (8)	168 (9)	**167 (9)**	175 (9)	175 (8)	173 (8)	174 (9)	173 (8)	**174 (8)**	< 0.01
% of HR_max_	84.5 (3.9)	85.9 (3.4)	84.3 (5.3)	86.3 (2.7)	86.0 (2.8)	86.0 (3.3)	**85.5 (3.6)**	90.6 (3.0)	90.7 (3.2)	89.3 (1.7)	89.9 (2.9)	89.4 (2.8)	**90.0 (2.7)**	< 0.01
Time ≥ 90% of HR (m:s)		13:50 (20:24)				14:00 (17:06)	**13:55 (18:28)**		28:17 (08:05)			22:53 (12:40)	**25:35 (10:47)**	< 0.01
PO (W·kg^−1^)	3.63 (0.32)	3.73 (0.33)	3.83 (0.39)	3.86 (0.49)	3.82 (0.54)	3.88 (0.39)	**3.79 (0.41)**	3.96 (0.63)	4.10 (0.62)	4.20 (0.52)	4.14 (0.54)	4.17 (0.60)	**4.11 (0.57)**	< 0.01
% of PO_V̇O2max_ (%)	63.3 (5.2)	64.8 (3.8)	66.5 (4.0)	66.8 (4.5)	65.8 (3.5)	67.4 (3.3)	**65.8 (4.2)**	68.3 (7.7)	70.5 (5.9)	72.4 (4.0)	71.4 (4.9)	71.8 (5.6)	**70.9 (5.8)**	< 0.01
[La^−^] (mmol·L^−1^)		2.8 (0.7)				3.1 (0.9)	**2.9 (0.8)**		7.3 (1.6)			7.6 (1.5)	**7.5 (1.5)**	< 0.01
PR (1–7)	2.8 (0.6)	2.9 (0.6)	2.9 (0.5)	3.1 (0.4)	3.2 (0.9)	2.9 (0.4)	**3.0 (0.6)**	3.0 (0.6)	3.0 (0.9)	3.3 (0.5)	3.3 (0.4)	3.2 (0.7)	**3.2 (0.6)**	0.04
RPE (6–20)	14.5 (0.3)	14.5 (0.4)	14.5 (0.3)	14.5 (0.2)	14.5 (0.2)	14.4 (0.2)	**14.5 (0.3)**	17.1 (0.3)	17.0 (0.4)	17.0 (0.5)	17.2 (0.3)	17.1 (0.5)	**17.1 (0.4)**	< 0.01
sRPE (0–10)	4.6 (1.1)	5.0 (0.8)	4.9 (0.8)	5.3 (0.7)	5.4 (0.8)	5.0 (0.7)	**5.0 (0.9)**	6.9 (1.3)	6.8 (0.8)	6.8 (1.1)	7.1 (1.3)	7.2 (0.9)	**6.9 (1.1)**	< 0.01

*Note:* Aerobic energy turnover, calculated total average aerobic energy turnover during the interval series at the second and fifth/sixth interval session; V̇O_2_, average oxygen consumption during the interval series at the second and fifth/sixth interval session; % of V̇O_2max_, average fraction of maximal V̇O_2_ during the interval series at the second and fifth/sixth interval session; time ≥ 90% of V̇O_2max_, time spent at or over 90% of V̇O_2max_ during the interval series at the second and fifth/sixth interval session; HR, average heart rate during the interval series; % of HR_max_, average fraction of maximal HR during the interval series; time ≥ 90% of HR_max_, time spent at or over 90% of HR_max_ during the interval series; PO, average power output during the interval series; % of PO_V̇O2max_, average fraction of maximal 1‐min incremental power output at the V̇O_2max_ test during the interval series; [La^−^], average blood lactate concentration measured 1 min after each interval series at the second and fifth/sixth interval session; PR, average rating of perceived readiness for the upcoming interval series reported 45‐s before each interval series; RPE, average rating of perceived exertion reported immediately after each interval series; sRPE, session rate of perceived exertion reported 10 min after each interval session. Bold values are averages across the respective interval sessions in both blocks. *p* ≤ 0.05 indicates a significant difference in average values between the MIT and HIT.

Following both interval blocks, a 6‐day recovery period was implemented. Day 1: complete rest; day 2: either rest or 20–40 min of low‐intensity exercise; day 3: 0.5–1.5 h of low‐intensity exercise; day 4: 20 min of low‐intensity exercise, 2 × 5‐min MIT, a 1‐min high‐intensity work interval with progressively increasing intensity, and 10 min of low‐intensity exercise; day 5: rest; day 6: 20 min of low‐intensity exercise, 2 × 5‐min MIT, 3 × 1‐min HIT with progressively increasing intensity, and 10 min of low‐intensity exercise.

The interval blocks were performed during the cyclists' preparatory period, so the cyclists performed their regular training before initiating the MIT block and between the two interval blocks. Participants reported their training during the 2‐week period preceding the interval blocks and during each block (Table 2). Endurance training intensity was categorized using a five‐zone scale based on the percentage of average HR during a 40‐min maximal cycling trial conducted at baseline (Hunter and Coggan 2010). Training impulse (TRIMP) scores were calculated by multiplying the accumulated duration in each intensity zone by a multiplier specific to that zone (e.g., 1 min in zone 1 equated to 1 TRIMP, 1 min in zone 2 equated to 2 TRIMP, and so forth) (Lucia et al. 2003).

**TABLE 2:** Training load during the 2‐week period prior to each block and during the 2‐week period with the moderate‐intensity interval training (MIT) block and subsequent active recovery period, and the high‐intensity interval training (HIT) block and subsequent active recovery period.

	Before MIT block	MIT block and active recovery	Before HIT block	HIT block and active recovery
Zone 1 (< 55% of PO_40min_; h:m)	07:58 (06:38)	04:43 (03:57)^*^	07:16 (06:26)	04:04 (02:59)^*^
Zone 2 (56%–75% of PO_40min_; h:m)	05:53 (04:16)	04:55 (02:20)	09:27 (06:06)^#^	04:43 (02:27)*
Zone 3 (76–90 of PO_40min_; h:m)	02:22 (00:58)	03:58 (01:19)^*^	04:41 (03:00)^#^	02:22 (00:51)* ^,^ ^#^
Zone 4 (91%–105% of PO_40min_; h:m)	02:25 (01:12)	05:44 (01:45)^*^	02:25 (01:17)	03:31 (00:49)* ^,^ ^#^
Zone 5 (> 106% of PO_40min_; h:m)	01:12 (01:48)	00:32 (00:31)^*^	00:30 (00:19)^#^	01:12 (01:08)* ^,^ ^#^
Total TRIMP	2550 (896)	3124 (366)^*^	3141 (1317)^#^	2442 (447)* ^,^ ^#^
Heavy resistance training (h:m)	00:53 (01:13)	00:09 (00:23)^*^	00:54 (01:23)	00:05 (00:16)^*^
Core training (h:m)	00:35 (00:46)	00:16 (00:23)^*^	00:11 (00:23)^#^	00:12 (00:27)
Total training (h:m)	20:58 (07:21)	20:17 (02:55)	24:54 (10:10)^#^	16:09 (03:54)* ^,^ ^#^
Feeling legs (1–9)	4.8 (0.8)	4.7 (0.7)	4.5 (0.9)	4.4 (0.7)

Abbreviation: TRIMP, training impulse.

^*^ Significantly different from the 2‐week period before the given block (*p* ≤ 0.05).

^#^ Significant difference between blocks (*p* ≤ 0.05).

### 2.4 Exercise Testing Procedures

For each participant at the first test, the last three meals, fluid intake, and caffeine intake prior to testing were recorded and replicated in all subsequent tests. Additionally, their nutritional energy intake during testing was noted and replicated during subsequent tests. All tests were performed at the same time of the day (± 2 h) for each participant.

### 2.5 Peak Isokinetic Unilateral Knee‐Extension Torque

Exercise testing was initiated with a 7‐min standardized cycling warm‐up. Maximal isokinetic unilateral knee‐extension torque was thereafter assessed at two angular speeds (60° and 240° per second) for a randomized leg using a dynamometer (Humac Norm, CSMi, Stoughton, MA, USA) as described in Mølmen et al. (2025). The leg used, seat position, and general settings were noted and replicated at the following tests for each participant to ensure consistency.

### 2.6 Prolonged Cycling Test

**FIGURE 2:**
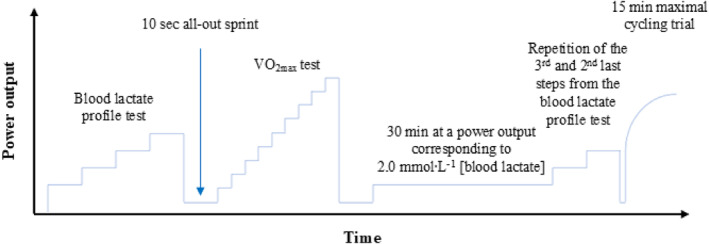
Overview of the prolonged cycling test. The protocol consisted of a number of different tests all conducted in continuum (1) a blood lactate profile test, (2) 5 min of active recovery, (3) a 10 s seated all‐out sprint, (4) 5 min of active recovery, (5) an incremental maximal oxygen uptake (V̇O_2max_) test, (6) 5 min of active recovery, (7) 30 min at a power output corresponding to 2.0 mmol·L^−1^ [blood lactate], repetition of the third and second last 5.5‐min step from the blood lactate profile, (8) 1 min of rest, and (9) a 15 min maximal cycling trial. The overall aim of the applied test battery was to evaluate exercise performance in a realistic manner while at the same time taking advantage of the controlled settings provided by an exercise laboratory.

Following 5 min of rest, a prolonged cycling test battery was initiated (Figure 2) (Mølmen et al. 2025). The test was performed on a Lode Excalibur Sport bicycle ergometer (Groningen, The Netherlands), which was adjusted according to individual preferences and replicated in all subsequent tests. All tests were conducted under similar environmental conditions (17.7 (1.4) °C), with airflow of 2–3m·s^−1^ directed toward the participants' frontal surface.

For both women and men, the blood lactate profile test started with 5 min of cycling at 125 W (175 W if self‐estimated lactate threshold > 325 W) and continued with 50 W increases every 5.5 min. With 30 s left of each 5.5‐min bout, capillary blood was sampled from the fingertip and analyzed for [La^−^]. When a [La^−^] of ≥ 2 mmol·L^−1^ was reached, the 5.5‐min bouts continued to increase with 25 W until a [La^−^] of ≥ 4 mmol·L^−1^ was attained. V̇O_2_ and RER were measured from 2.5 to 5 min in each bout, with a sampling time of 30 s (Vyntus CPX, Jaeger‐CareFusion, UK). From the blood lactate profile test, the power output corresponding to 4 mmol·L^−1^ [La^−^] (PO_4mmol_) and the fractional utilization of V̇O_2max_ at PO_4mmol_ (% of V̇O_2max_ at PO_4mmol_) were calculated by plotting [La^−^] as a function of power output and % of V̇O_2max_ at each 5.5‐min bout (linear regression between data points).

Following 5 min of active recovery, a seated sprint was performed to measure 10‐s maximal mean power output (PO_10sec_), with the torque factor set to 0.70 and 0.85 for female and male participants, respectively. After an additional 5 min of active recovery, an incremental test for determination of V̇O_2max_ was initiated at 200/250 W for males and 160 W for females with 25/20 W increases every minute, respectively, until exhaustion, defined as cadence < 60 revolutions per min. V̇O_2max_ was defined as the mean of the 12 highest consecutive 5‐s measurements. PO_V̇O2max_ was defined as the last minute mean load of the V̇O_2max_ test, while maximal HR (HR_max_) was defined as the highest value during the test.

After the V̇O_2max_ test, participants rested for 5 min before they initiated 30 min of cycling at a power output corresponding to 2 mmol·L^−1^ [La^−^] as calculated from the blood lactate profile. Thereafter, the third and second last 5.5‐min step from the blood lactate profile test was repeated in the semi‐fatigued state. During these repetitions, HR, V̇O_2,_ and RER were measured with a sampling time of 30‐s from 2.5 to 5 min at each bout. Steady‐state V̇O_2_ and RER were used to calculate gross efficiency (GE) at the third and second last step from the blood lactate profile in both the fresh and semi‐fatigued state. GE was defined as the ratio between metabolic power input (PI) and mechanical power output (PO; GE = (PI·PO^−1^)·100). PI was determined using the measured V̇O_2_ and RER at the given power output and the energetic equivalent of O_2_ (Péronnet and Massicotte 1991) according to the constants proposed by Kipp et al. (2018); PI = V̇O_2_ L·s^−1^ · (4840 J·L^−1^ · RER + 16890 J·L^−1^).

Following 1 min of recovery participants performed a 15‐min maximal cycling trial during which they were instructed to aim for their highest possible mean power output and adjusted the power output themselves. Performance was measured as the average power output during the test (PO_15min_), and V̇O_2_ was continuously measured during the test to calculate the fractional utilization of V̇O_2max_ (% of V̇O_2max_ at PO_15min_).

### 2.7 Statistics

Descriptive data are presented as means with standard deviations (mean (SD)). Results were considered statistically significant if *p* ≤ 0.05. All data analysis was performed in R (version 4.4.2) (R Core Team 2023). To increase the statistical power for performance‐related measures, an endurance performance index based on the main performance indicators (PO_V̇O2max_, PO_4mmol_, and PO_15min_) was calculated as the average of the given indicators after normalization (*x*_*i*_ · max(*x*)^−1^ where *x*_*i*_ is a single observation for one performance indicator and max(x) is the maximum value observed across all participants for the given indicator) (Solli et al. 2025; Odden et al. 2024). Differences in training load during interval sessions between blocks and differences in overall training load during the 2 weeks preceding both interventions and the 2‐week intervention periods were examined using linear mixed models fitted with the training variable of interest as the dependent variable, training period as the fixed effect, and the subject indicator as a random effect (lme4 package) (Bates et al. 2015). The pairwise comparisons of interest were derived from the linear mixed models (emmeans package) (Lenth 2025). Differences in pre to post change scores between blocks were investigated using linear mixed models fitted with the outcome of interest as the dependent variable, the interaction between block and time as a fixed effect, the given baseline value as a covariate, and the participant indicator as a random effect. To examine pre‐to‐post differences within blocks, pairwise comparisons of the estimated marginal means were derived from the linear mixed models. To interpret the practical significance of differences between groups, Cohen's d effect size (ES) was calculated using percentage changes (%‐point changes for GE and % of V̇O_2max_) (effsize package) (Torchiano 2016). The scale proposed by Rhea (Rhea 2004) for highly trained subjects was used to interpret the magnitude of the treatment effect: 0.0–0.24, trivial; 0.25–0.49, small; 0.5–1.0, moderate; and > 1.0, large. Relationships between training variables and training adaptations in the respective blocks were explored using multiple linear regression models, fitted with the change scores of the outcome variable of interest as the dependent variable, the training variable of interest as the fixed effect, and baseline values and change in body mass as covariates (R stats package) (R Core Team 2023). For the regression models, estimated scores (indicative of the theoretical change in the dependent variable for each unit change in the independent variable), along with 95% confidence intervals, *p*‐values, and *R* ^2^_adjusted_, are reported.

## 3 Results

**FIGURE 3:**
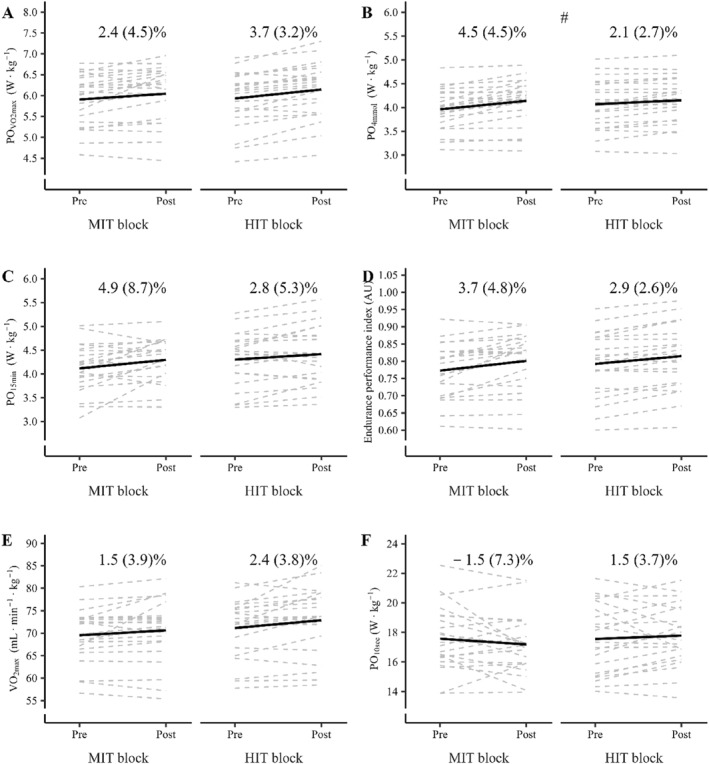
Individual data points (dotted lines) and mean values (solid lines) for (A) maximal 1‐min incremental power output during the maximal oxygen uptake (V̇O_2max_) test (PO_V̇O2max_), (B) power output at 4 mmol·L^−1^ lactate concentration (PO_4mmol_), (C) maximal mean power output during the 15 min cycling trial (PO_15min_), (D) the endurance performance index, (E) V̇O_2max_, and (F) mean power output during the 10 s all‐out sprint (PO_10sec_), before (pre) and after (post) the moderate‐intensity interval training (MIT) block and the high‐intensity interval training (HIT) block. The values presented in each panel represent the mean (SD) percentage change in the variable of interest for the MIT and HIT blocks, respectively. ^#^Absolute change significantly differs between blocks (*p* ≤ 0.05).

Pre and post values for all investigated outcomes in the MIT block and HIT block are presented in Table 3, with individual and group responses for selected outcomes illustrated in Figure 3.

**TABLE 3:** Physiological responses to the moderate‐intensity interval training (MIT) block and the high‐intensity interval training (HIT) block, respectively.

	MIT block		HIT block		Block × time *p*	Effect size
	Pre	Post	Pre	Post		
Body mass (kg)	70.8 (7.9)	71.0 (8.0)	71.2 (8.4)	71.4 (8.2)	0.93	−0.01
PO_V̇O2max_ (W·kg^−1^)	5.91 (0.62)	6.05 (0.67)*	5.93 (0.66)	6.15 (0.66)*	0.27	−0.34
PO_4mmol_ (W·kg^−1^)	3.96 (0.43)	4.14 (0.47)*	4.07 (0.5)	4.15 (0.50)*	0.03	0.64
PO_15min_ (W·kg^−1^)	4.12 (0.51)	4.30 (0.45)*	4.30 (0.57)	4.42 (0.61)*	0.44	0.29
Endurance performance index (arbitrary value, 0–1)	0.773 (0.081)	0.801 (0.084)*	0.792 (0.095)	0.815 (0.097)*	0.53	0.21
PO_10‐sec_ (W·kg^−1^)	13.51 (1.20)	13.28 (1.25)	13.64 (1.36)	13.82 (1.24)	0.08	−0.51
V̇O_2max_ (mL·min^−1^·kg^−1^)	69.5 (6.0)	70.6 (7.0)	71.1 (6.5)	72.9 (7.3)*	0.43	−0.23
% of V̇O_2max_ at PO_4mmol_ (%)	79.2 (3.8)	80.3 (3.3)*	79.6 (3.3)	78.8 (3.2)	0.02	0.68
% of V̇O_2max_ at PO_15min_ (%)	84.1 (5.4)	84.4 (3.5)	85.0 (4.2)	83.4 (3.8)*	0.06	0.52
GE 3^rd^ last step fresh (%)	19.6 (1.5)	19.9 (1.5)*	19.6 (1.4)	19.8 (1.5)	0.52	0.20
GE 3^rd^ last step tired (%)	18.9 (1.5)	19.3 (1.4)*	19.0 (1.3)	19.3 (1.4)*	0.59	0.16
GE 2^nd^ last step fresh (%)	19.9 (1.2)	20.2 (1.2)*	19.9 (1.3)	20.0 (1.2)	0.43	0.23
GE 2^nd^ last step tired (%)	19.4 (1.4)	19.6 (1.2)	19.3 (1.1)	19.5 (1.2)	0.79	−0.07
Peak isokinetic unilateral knee‐extension torque (Nm·kg^−1^)						
60°·s^−1^	2.78 (0.35)	2.63 (0.29)*	2.54 (0.29)	2.52 (0.34)	0.02	−0.61
240°·s^−1^	1.66 (0.20)	1.65 (0.21)	1.63 (0.25)	1.62 (0.24)	0.88	0.01

*Note:* PO_V̇O2max_, maximal 1 min power output during the maximal oxygen consumption test (V̇O_2max_ test); PO_4mmol_, power output at 4 mmol·L^−1^ blood lactate concentration ([La^−^]); PO_15‐min_, maximal average power output during the 15‐min cycling trial; % of V̇O_2max_ at PO_4mmol_, fractional utilization of V̇O_2max_ at PO_4mmol_; % of V̇O_2max_ at PO_15min_, fractional utilization of V̇O_2max_ during the 15 min cycling trial; PO_10sec_, mean power output during the 10‐s seated all‐out sprint; GE third last step fresh, gross efficiency measured at the third last step of the blood lactate profile in the fresh state, GE third last step tired, gross efficiency measured at the repeated third last step of the blood lactate profile in the tired state, GE second last step fresh, gross efficiency measured at the second last step of the blood lactate profile in the fresh state, GE second last step tired, gross efficiency measured at the repeated second last step of the blood lactate profile in the tired state. Values are mean (SD).

^*^ Significant difference from pre to post within block (*p* ≤ 0.05). Block × time *p* ≤ 0.05 indicates significant difference in absolute change between blocks.

### 3.1 Endurance Performance Measures

The endurance performance index improved in both the MIT block (0.028 (0.035) AU; *p* < 0.01) and HIT block (0.023 (0.020) AU; *p* < 0.01), with no difference observed between blocks (*block* × *time p* = 0.53). Similarly, PO_15min_ increased in both the MIT block (0.18 (0.31) W·kg^−1^; *p* < 0.01) and HIT block (0.12 (0.22) W·kg^−1^; *p* = 0.05), with no difference between blocks (*block* × *time p* = 0.44). Moreover, improvements in PO_V̇O2max_ did not differ between the MIT block (0.14 (0.26) W·kg^−1^; *p* < 0.01) and HIT block (0.22 (0.18) W·kg^−1^; *p* < 0.01; *block* × *time p* = 0.27). However, the improvement in PO_4mmol_ was greater in the MIT block (0.18 (0.17) W·kg^−1^; *p* < 0.01) than in the HIT block (0.08 (0.1) W·kg^−1^; *p* < 0.01; *block* × *time p* = 0.03). Changes in mean power output during the 10‐s sprint (PO_10sec_) did not differ between the MIT block (−0.23 (1.01) W·kg^−1^; *p* = 0.16) and HIT block (1.18 (0.47) W·kg^−1^; *p* = 0.28; *block* × *time* = 0.08).

### 3.2 Physiological Determinants of Endurance Performance

V̇O_2max_ increased within the HIT block (1.7 (2.8) mL·min^−1^·kg^−1^; *p* < 0.01), but not the MIT block (1.1 (2.7) mL·min^−1^·kg^−1^; *p* = 0.06), with no difference observed between blocks (*block* × *time p* = 0.43). Changes in % of V̇O_2max_ at PO_4mmol_ differed between blocks in favor of the MIT block (*block* × *time p* = 0.02), with an increase within the MIT block (1.2 (3.4) %‐points; *p* = 0.05) but not the HIT block (−0.9 (2.5) %‐points; *p* = 0.15). Changes in % of V̇O_2max_ at PO_15min_ did not differ between blocks (*block* × *time p* = 0.06), with no change within the MIT block (0.3 (3.6) %‐points; *p* = 0.66) and a reduction within the HIT block (−1.6 (3.6) %‐points; *p* = 0.03). Changes in GE in both the fresh and semi‐fatigued state did not differ between blocks (*p*‐values presented in Table 3). In the fresh state, the MIT block improved GE at the third (0.3 (0.6) %‐points; *p* = 0.01) and the second last step of the blood lactate profile (0.2 (0.5) %‐points; *p* < 0.01)_,_ while the HIT block did not improve GE at neither the third (0.2 (0.5) %‐points; *p* = 0.11) or second last step (0.2 (0.3) %‐points; *p* = 0.08). In the semi‐fatigued state, the MIT block improved GE at the third last step (0.4 (0.7) %‐points; *p* < 0.01) but not the second last step (0.2 (0.9) %‐points; *p* = 0.22), and the HIT block improved GE at the third last step (0.3 (0.4) %‐points; *p* = 0.03) but not the second last step (0.2 (0.4) %‐points; *p* = 0.11).

### 3.3 Muscle Strength Measures

Changes in peak isokinetic knee extension torque at the angular speed of 60°·s^−1^ differed between blocks (*block* × *time p* = 0.02), with a reduction within the MIT block (−0.15 (0.20) Nm·kg^−1^; *p* < 0.01) and no change within the HIT block (−0.03 (0.13) Nm·kg^−1^; *p* = 0.45). Peak isokinetic knee‐extension torque at 240°·s^−1^ did not display significant differences between (*p*‐values presented in Table 3) or within blocks (data not shown).

### 3.4 The Effect of Oxygen Uptake During the Intervals on Training Adaptations

**FIGURE 4:**
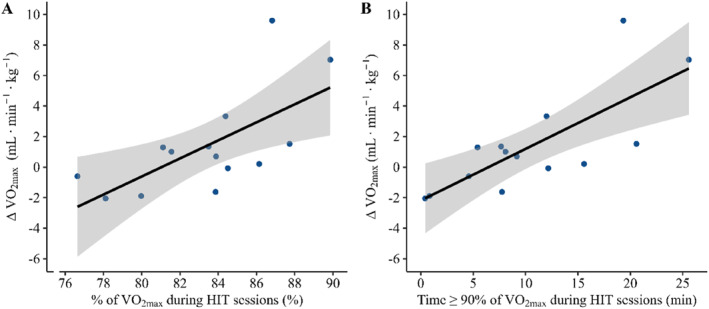
Multiple linear regression of (A) the average fraction of maximal oxygen uptake (% of V̇O_2max_) and (B) time spent ≥ 90% of V̇O_2max_ during work intervals at the second and fifth interval session in the high‐intensity interval training (HIT) block related to changes in V̇O_2max_ when controlling for baseline values, change in body mass, and sex. Individual data‐points for the participants in the HIT block and regression slopes (solid lines) with 95% confidence intervals (light gray areas) are shown.

For the HIT block, there was a positive relationship between % of V̇O_2max_ during work intervals at the second and last HIT session and changes in V̇O_2max_ (estimate = 0.78 [0.29, 1.27] mL·min^−1^·kg^−1^ theoretical increase for each %‐point higher % of V̇O_2max_ during work intervals; *p* < 0.01; *R* ^2^_adjusted_ = 0.45; Figure 4A). Additionally, a positive relationship was observed between time spent ≥ 90% of V̇O_2max_ during work intervals in the HIT block and changes in V̇O_2max_ (estimate = 0.41 [0.23, 0.59] mL·min^−1^·kg^−1^ theoretical increase for each additional min spent ≥ 90% of V̇O_2max_ during work intervals; *p* < 0.01; *R* ^2^_adjusted_ = 0.65; Figure 4B). In the MIT block, no relationships were observed between V̇O_2max_ changes and % of V̇O_2max_ (estimate = 0.02 [−0.29, 0.32] mL·min^−1^·kg^−1^; *p* = 0.90; *R* ^2^_adjusted_ = −0.10) or time spent ≥ 90% of V̇O_2max_ during work intervals (estimate = 0.09 [−0.08, 0.26] mL·min^−1^·kg^−1^; *p* = 0.27; *R* ^2^_adjusted_ = 0.05). In both blocks, no relationships were observed for other outcome variables (data not shown).

## 4 Discussion

The main findings from the present study align with the initial hypothesis, demonstrating that both a MIT and a HIT block effectively improve surrogate measures of endurance performance, with minor differences between blocks. Partly in support of our hypothesis, there were indications of work‐intensity specific adaptations between the blocks. Specifically, the MIT block induced a larger improvement in PO_4mmol_ compared to the HIT block (4.5% vs. 2.1%, respectively), while the HIT block displayed a moderate ES for change in PO_10sec_ compared to the MIT block (1.5% vs. −1.5%, respectively) although this difference was not statistically significant (*p* = 0.08). Furthermore, in line with the initial hypothesis, time spent ≥ 90% of V̇O_2max_ during the HIT block was positively related to changes in V̇O_2max_, while no relationship was observed during the MIT block, suggesting that adaptational mechanisms may differ between blocks.

Previous 1‐week block training studies in well‐trained endurance athletes have predominantly focused on the effects of HIT, reporting ∼3%–5% improvements in PO_V̇O2max_ (Rønnestad et al. 2021; Rønnestad and Vikmoen 2019; Rønnestad, Bjerkrheim, et al. 2022; Solli et al. 2025). For the first time, this study demonstrates that similar performance improvements can be induced by a 1‐week MIT block characterized by a total work interval duration almost twice that of a HIT block, but at a lower exercise intensity. The latter was evident by the MIT block displaying lower values than the HIT block for mean HR (∼85 vs. ∼90% of HR_max_), mean blood lactate concentration (∼3 vs. ∼7 mmol·L^−1^), mean RPE (∼14.5 vs. ∼17.0), and session RPE (∼5 vs. ∼7). The finding of similar improvements in PO_15min_ and the calculated endurance performance index across blocks agrees with the suggestion that MIT can serve as a useful strategy for enhancing endurance performance by providing a notable aerobic training stimulus sustained for a substantial duration (Burnley et al. 2022). Accordingly, studies comparing training adaptations during ∼ moderate‐intensity training with HIT, applying two to three times longer durations with moderate‐intensity exercise than HIT (which is similar to the training performed by elite athletes), observe similar training adaptations between the two intensities (Seiler et al. 2013; Sylta et al. 2017; Jarstad and Mamen 2019). Collectively, this indicates that also in well‐trained endurance athletes, the adaptational signal of exercise training is affected by both the exercise intensity and the accumulated duration of work intervals in an integrated manner. Notably, when total energy turnover is matched, HIT is observed to induce superior training adaptations compared to moderate‐intensity exercise training (Helgerud et al. 2007).

It can be argued that for these highly trained athletes, it is of interest to discuss trends in the data set and not only the differences that reached statistical significance, especially when considering the short length of the intervention period. However, the following discussion of trends must be interpreted in light of the relatively large drop‐out rate and its associated risk of biased outcomes, despite a final sample of *n* = 22, which is relatively high given the training status of the participants. In accordance with the training principle of specificity, there were indications of work intensity‐specific adaptations between the two blocks. The HIT block induced a numerically larger increase than the MIT block in PO_10sec_ (1.5% vs. −1.5%, respectively) with an accompanied moderate ES in favor of HIT compared to MIT. The numerically larger increases in PO_V̇O2max_ (3.7% vs. 2.4%, respectively) gave a small ES in favor of HIT compared to MIT although no statistically significant difference in changes were observed between the blocks. These ESs could be related to the work interval intensity being closer to PO_V̇O2max_ during HIT than MIT. In the same manner, the MIT block induced a larger improvement in PO_4mmol_ than HIT (4.5% vs. 2.1%, respectively) with a moderate ES, which is an exercise intensity closer to MIT than HIT, suggesting that the use of this work intensity triggers muscle‐specific adaptations, including clearance of lactate (Philp et al. 2008). The greater improvement in PO_4mmol_ observed in the MIT block compared to the HIT block may be partly attributed to the larger increase in % of V̇O_2max_ at PO_4mmol_. However, this change in % of V̇O_2max_ at PO_4mmol_ may also be influenced by the numerically smaller V̇O_2max_ increase in the MIT block, as this variable is a ratio where V̇O_2max_ serves as the denominator. Thus, care must be taken when interpreting changes in such derived variables across interventions with differing V̇O_2max_ responses. Collectively, it appears that the MIT and HIT block target slightly different determining factors of endurance performance and therefore end up with similar improvements in the primary endurance performance measure, PO_15min_, and in the calculated endurance performance index. These findings align with the ones of a study on moderately trained soldiers, where both a 1‐week block with either HIT or continuous running at moderate‐intensity enhanced performance (Borszcz et al. 2022). That study also indicated work‐intensity specific adaptations in terms of favoring moderate‐intensity training on threshold adaptations and HIT on V̇O_2max_ adaptations (Borszcz et al. 2022). Similarly, studies of trained cyclists and runners have also observed tendencies of training intensity‐specific adaptations when the moderate‐intensity group performs a larger total work duration than the HIT group (Seiler et al. 2013; Sylta et al. 2017; Jarstad and Mamen 2019). Both HIT and MIT blocks induced modest improvements in given GE outcomes, with no group differences. This was surprising considering that in general, work economy is thought to be relatively stable, with elite cyclists often showing no changes in work economy over extended periods (Lucía et al. 2000; Sassi et al. 2008).

The present study was not specifically designed to investigate the underlying mechanisms behind training adaptations. However, it is noteworthy that both % of V̇O_2max_ and time spent ≥ 90% of V̇O_2max_ during work intervals were related to V̇O_2max_ improvements in the HIT block but not in the MIT block. This agrees with previous observations that time ≥ 90% of V̇O_2max_ during HIT exercise is beneficial for training adaptations (Rønnestad, Bjerkrheim, et al. 2022; Odden et al. 2024; Turnes et al. 2016) and supports the role of time spent ≥ 90% of V̇O_2max_ for training adaptations during HIT interventions. Speculatively, cellular stress appears to increase in proportion to exercise intensity (Egan and Zierath Juleen 2013; MacInnis and Gibala 2017), and it has been suggested that the adaptational stimulus is exercise intensity‐dependent up to V̇O_2max_ (Midgley et al. 2006). Therefore, it can be hypothesized that exercising ≥ 90% of V̇O_2max_ during the HIT block imposes a substantial stress on the oxygen transport and utilization chain, triggering both peripheral and central adaptations associated with improved PO_4mmol_ and PO_V̇O2max_. Indeed, it has been observed that HIT block periodization induces a moderate increase in hemoglobin mass compared to traditional exercise training in cyclists (Rønnestad et al. 2014). Additionally, peripheral adaptations like capillarization, as well as increased size, function, and number of mitochondria (Holloszy and Coyle 1984), along with improvements in cardiorespiratory factors, could have played a role for the enhanced performance following the HIT block (Wen et al. 2019; Midgley and Mc Naughton 2006; Buchheit and Laursen 2013).

The lack of correlation between time ≥ 90% of V̇O_2max_ and training adaptations in the MIT block is indicative of slightly different adaptational signal mechanisms between the blocks. The different manipulation of work interval intensity and duration likely led to slightly different acute stimuli between blocks (Hoppeler et al. 2011), yet these differences may still result in quite similar training adaptations. Speculatively, MIT may induce capillary growth through prolonged intracellular increases in calcium ions and shear stress in the capillaries (Ishan et al. 2014; Hoier and Hellsten 2014) potentially enhancing the oxygen diffusion capacity from blood to muscles. Similarly, MIT and HIT likely trigger specific signals for mitochondrial biogenesis, albeit with slightly different mechanisms, both ending up with functional adaptations (MacInnis et al. 2019; Bishop et al. 2019). However, the short time frame of the intervention (∼2 weeks) and no invasive measurements makes it impossible to conclude on mechanisms for either blocks.

It is important to note that while this study holds methodological advantages by performing both blocks within the same cohort of well‐trained cyclists, this study is not a randomized cross‐over study. All cyclists performed the MIT block first (in October/November) and following a wash‐out period with regular training they undertook the HIT block (in January). This lack of randomization might have favored the MIT block due to the slightly lower pre‐values observed for PO_4mmol_, V̇O_2max_, and PO_15min_, indicating a larger potential for adaptation. However, it should be noted that we have used a statistical approach accounting for potential baseline differences between blocks. Previous training interventions comparing high‐ and moderate‐intensity exercise have matched for either total work or energy consumption (i.e., isoenergetic (Helgerud et al. 2007)), or effort with two to three times longer work intervals in ∼MIT compared to HIT (i.e., isoeffort (Seiler et al. 2013; Sylta et al. 2017)). The comparison of exercise stimulus in the present study was not specific to any of the mentioned approaches. Instead, it was guided by RPE to mimic the typical differences in effort between MIT and HIT sessions (e.g., less exertion but longer duration during MIT compared to HIT). The approach was chosen to circumvent the inherent issues associated with exercise prescriptions based on fixed percentages of V̇O_2max_, PO_V̇O2max_, HR_max_, and lactate threshold power output (Iannetta et al. 2020; Jamnick et al. 2020). It has been suggested that there exists a threshold in fatigue development depending on whether exercise is performed just below or just above an exercise intensity around the maximal metabolic steady state (often measured as 4 mmol·L^−1^ [La^−^]) (Burnley et al. 2012). In this context, the ∼50% higher total aerobic energy turnover and larger training volume in the MIT block (exercising at ∼3 mmol·L^−1^ [La^−^]) compared to the HIT block induced a reduction in peak isokinetic knee‐extension torque at 60°·sec^−1^, but not at 240°·sec^−1^, making it unclear whether the contractile recovery status differed after the two blocks. Still, the study experienced a considerable dropout rate. Although excessive training load could potentially contribute to increased susceptibility to illness, in this case, it is more likely attributable to outbreaks of respiratory tract infections during the late autumn (October/November), as participants trained and attended school together. The consequence of this outbreak was that the majority of dropouts occurred in the MIT block (*n* = 7) and during the subsequent wash‐out period with usual training (*n* = 12). That being said, 22 participants completed both training blocks.

In the present study, the MIT and HIT blocks were not compared to a time‐matched period of regular training, preventing direct comparisons of these blocks and regular training. However, previous HIT (Rønnestad, Bjerkrheim, et al. 2022; Solli et al. 2025; Breil et al. 2010; Clark et al. 2014) and MIT (Mølmen et al. 2025) block interventions, similar to the present, have observed greater adaptations in the block group compared to control groups that continued regular training. Additionally, when athletes are highly trained and familiar with the testing protocol (as in the present study), it is uncommon to observe improvements after only 2 weeks of regular training. For decades, there has been a debate over whether accumulating shorter durations at intensities close to V̇O_2max_ or accumulating longer durations at a lower intensity (e.g., around lactate threshold) yields superior training adaptations (Åstrand and Rodahl 1986). The findings of the present study suggest that rather than focusing on which approach is superior, it may be more relevant to recognize the distinct advantages and specificities of both MIT blocks and HIT blocks. We argue that in well‐designed training regimens, both MIT and HIT should be integrated along with low‐intensity exercise to maximize endurance adaptations.

In conclusion, both a 1‐week MIT block (lower exercise intensity but longer work intervals) and a 1‐week HIT block (higher exercise intensity but shorter work intervals) effectively improved endurance performance determinants as well as the primary endurance measure, PO_15min_. The observed changes indicate some work intensity‐specific adaptations, with time spent ≥ 90% of V̇O_2max_ being related to training adaptations in the HIT block but not in the MIT block.

## Consent

Prior to inclusion, all participants provided written informed consent to participate in the study.

## Conflicts of Interest

The authors declare no conflicts of interest.

## Data Availability

The data collected and analyzed during the current study are available from the corresponding author upon reasonable request.
